# Experiences of Indian Health Workers Using WhatsApp for Improving Aseptic Practices With Newborns: Exploratory Qualitative Study

**DOI:** 10.2196/medinform.8154

**Published:** 2018-03-01

**Authors:** Parika Pahwa, Sarah Lunsford, Nigel Livesley

**Affiliations:** ^1^ University Research Co, LLC Delhi India; ^2^ EnCompass LLC Chevy Chase, MD United States

**Keywords:** quality improvement, mobile apps, communication, patient care team

## Abstract

**Background:**

Quality improvement (QI) involves the following 4 steps: (1) forming a team to work on a specific aim, (2) analyzing the reasons for current underperformance, (3) developing changes that could improve care and testing these changes using plan-do-study-act cycles (PDSA), and (4) implementing successful interventions to sustain improvements. Teamwork and group discussion are key for effective QI, but convening in-person meetings with all staff can be challenging due to workload and shift changes. Mobile technologies can support communication within a team when face-to-face meetings are not possible. WhatsApp, a mobile messaging platform, was implemented as a communication tool by a neonatal intensive care unit (NICU) team in an Indian tertiary hospital seeking to reduce nosocomial infections in newborns.

**Objective:**

This exploratory qualitative study aimed to examine experiences with WhatsApp as a communication tool among improvement team members and an external coach to improve adherence to aseptic protocols.

**Methods:**

Ten QI team members and the external coach were interviewed on communication processes and approaches and thematically analyzed. The WhatsApp transcript for the implementation period was also included in the analysis.

**Results:**

WhatsApp was effective for disseminating information, including guidance on QI and clinical practice, and data on performance indicators. It was not effective as a platform for group discussion to generate change ideas or analyze the performance indicator data. The decision of who to include in the WhatsApp group and how members engaged in the group may have reinforced existing hierarchies. Using WhatsApp created a work environment in which members were accessible all the time, breaking down barriers between personal and professional time. The continual influx of messages was distracting to some respondents, and how respondents managed these messages (eg, using the silent function) may have influenced their perceptions of WhatsApp. The coach used WhatsApp to share information, schedule site visits, and prompt action on behalf of the team.

**Conclusions:**

WhatsApp is a productive communication tool that can be used by teams and coaches to disseminate information and prompt action to improve the quality of care, but cannot replace in-person meetings.

## Introduction

Interprofessional teamwork is an essential component of effective quality improvement (QI) in health care [1]. Health care staff must collaborate to make workflows and processes of care more efficient and improve patient outcomes. Successful teamwork in QI requires functional communication structures in which team members can participate [2]. Inclusive leadership and communication can create an environment in which staff feel valued, appreciated, and empowered to contribute to improvement efforts [3]. Communication failures in interprofessional teams can be attributed to different communication styles by cadre, hierarchical structures, and a culture in which mistakes are perceived as personal failings [4]. With high patient loads and conflicting schedules, it can be a challenge to find time for face-to-face communication with all or most of a QI team at regular intervals. Mobile technologies present an opportunity for team members to remain connected in between or in lieu of in-person meetings.

mHealth, “medical and public health practice supported by mobile devices” [5], has been applied in the health sector in low- and middle-income countries (LMICs) predominantly to educate and promote behavior change among patients. mHealth approaches have also been applied to medical imaging, collecting and transmitting patient-level data, and providing support to health workers in clinical tasks, communication with patients, and supply chain management [6]. A recent framework on mHealth as a health systems strengthening tool presented 12 common applications of mHealth, including to improve adherence to clinical protocols [7]. Another systematic review identified five uses of mobile technologies by frontline workers in LMICs [8]. Notably absent in the research from LMICs is the use of mobile platforms to support communication among health workers as a mechanism for improving care systems and processes.

WhatsApp is a messaging platform that allows users to send text messages, photographs, documents, and videos and make phone calls using a smartphone. The app allows for group chats with up to 256 members. There are an estimated one billion WhatsApp users worldwide, with about 160 million of those in India [9]. In health care in high-resource settings, WhatsApp has been employed as a means of communication in laboratory services [10] and emergency surgery [11], and in LMICs, it has been used to facilitate supervision of community health workers [12].

In spite of its use in health care, we found no published research on the application of WhatsApp or similar platforms to facilitate the work of QI teams in any setting. We sought to explore how a QI team in one Indian hospital communicated with each other and with a coach via WhatsApp while implementing modern improvement methods to improve adherence to aseptic protocols in the neonatal intensive care unit (NICU).

## Methods

### Study Site

The study hospital is a tertiary-level care hospital in Delhi providing free services to a population of 300,000 with nearly 7000 deliveries every year. The 15-bed NICU has a bed occupancy rate of 50% and is staffed by 5 pediatricians, 6 general doctors, 12 nurses, and 2 paramedical staff who provide round-the-clock services for an average of 120 newborn admissions per month.

The hospital had experience implementing modern QI methods with support from the United States Agency for International Development Applying Science to Strengthen and Improve Systems (USAID ASSIST) Project in the gynecology department, but this was the first QI activity implemented in the NICU. The positive impact of QI implementation in other departments and results from other QI projects in local hospitals were instrumental in motivating the NICU staff to take up this project. The NICU improvement team consisted of 21 staff, including 1 head of department, 1 senior specialist, 3 senior residents, 6 junior residents, 2 senior staff nurse, 1 sister in charge, 5 staff nurse, and 2 technicians. In total, 12 team members joined when the team was formed and formed the core team; the remaining 9 participated in the improvement activity as they were able.

The NICU team observed that the QI approach involved the following 4 steps: (1) forming a team to work on a specific aim, (2) analyzing the reasons for current poor performance, (3) developing changes that could improve care and testing these changes using plan-do-study-act (PDSA) cycles, and (4) implementing successful changes to sustain improvements. Analysis of unit data revealed that babies’ length of stay was increasing due to nosocomial infections, as aseptic protocols during intravenous (IV) procedures were not properly followed. In May 2016, the team began a QI activity on following asepsis protocol while performing IV procedures and collecting data regarding number of blood draws done on daily basis.

The QI team tested and implemented the following changes: prearranging blood sampling trays with all necessary items required to perform aseptic procedures; preparing a checklist to evaluate performance; and establishing a cardboard dropbox into which observers deposited completed checklists to make the evaluation process anonymous. The team continued to do PDSA cycles to eliminate unnecessary steps in the blood sampling process to make it simpler and adaptable to hospital staff. Within 12 weeks of starting the improvement activity, the team followed aseptic protocols in 80% of the blood samples taken and decided to expand their activities to improve central lines procedures.

Over the course of 18 weeks, the QI team received 8 in-person coaching visits from an external advisor with 3 years of experience in helping health workers use QI approaches. The coach was also in the WhatsApp group and engaged in regular communication. Not all in-person visits were equally fruitful due to medical emergencies or scheduling that prevented the team from coming together. The coach guided the team through the learning process of collecting, analyzing, and interpreting data to identify gaps in quality and generating changes to test to address those gaps.

### Study Design

An exploratory, qualitative case study design was used to examine the role of WhatsApp in QI team communication and coaching. Ten team members were purposively selected for interviews to represent different cadres and tenures at the facility (Table 1). The coach who supported the team was also included in the interview sample to present an alternate perspective on WhatsApp for coaching and improvement.

**Table 1:** Respondents by title, sex, and role in quality improvement team. NICU: neonatal intensive care unit.

Pseudonym	Designation	Sex	Role in quality improvement team
Dr Manish	Senior Consultant, NICU	Male	Team leader
Dr Vijay	Senior Resident	Male	Team member
Dr Arun	Consultant	Male	Associate team leader
Dr Nisha	Senior Resident	Female	Team member
Dr Rajiv	Junior Resident	Male	Team member: took the lead in finalizing the standard operating procedure for asepsis protocol; used flowchart as tool for identifying the process
Anita	Senior Staff Nurse	Female	Team member: prepared checklist for evaluation and dropbox; main communicator for passing on all information to the unit people; active member on WhatsApp
Manjeet	Senior Staff Nurse	Male	Team member
Seema	Staff Nurse	Female	Team member: prepared sample collecting tray; was not on WhatsApp despite being an active team member
Kajal	Staff Nurse	Female	Team member
Raman	Technician	Male	Team member: responsible for data collection
Anay	Coach	Male	Coach

### Data Collection

Semistructured interviews of team members were conducted by the first author. The coach who provided support to the team was interviewed by the second author. Questions on communication methods and the use of WhatsApp were part of a longer interview on team-based QI. Interviews were conducted at the hospital in Hindi. Audio recordings were transcribed and translated into English for analysis. In all, 18 weeks of the WhatsApp transcript (May-August 2016) were accessed, including images and files that were shared in the group. Communication in the WhatsApp group was predominantly in English.

### Data Analysis

Interviews were coded using in vivo, process, and structural coding strategies. An initial coding scheme was developed by the first two authors; it was refined by the second author and applied to all interviews. Codes were then aggregated into categories and themes during discussions among all authors. Frequency of type and form of messages (quantification) of the WhatsApp transcript was conducted. Analysis was done using NVivo 11 (QSR International, Burlington, MA, USA).

Ethics approval was granted by University Research Co., LLC (USA). The purpose and procedures of this research were explained to all respondents, and informed consent was obtained. Pseudonyms have been used to protect confidentiality.

## Results

### Setting Up and Managing a WhatsApp Group

The WhatsApp group was established by Dr Manish, team leader, who was having difficulty scheduling a meeting to share a new standard operating procedure and thought a communications app could be of use, especially considering staff had already used mobile technology to communicate among themselves. Dr Manish had the perception that WhatsApp was universally used among the staff, an opinion shared by 3 other respondents, so it would be easy to employ the app in the workplace:

I think everyone is using WhatsApp for everything. So I thought let’s make a WhatsApp group and there I will ask about project details. I made a group.

The real or imagined hierarchy in the facility influenced who was added to the WhatsApp group. Dr Manish recognized some hesitation on behalf of the nurses to share their mobile numbers with their superiors so they could be added to the group:

Yes I took help; I didn’t have contacts of all. Anita was there so I had to ask her to add few numbers. Nurses are not so comfortable to give their numbers to everyone especially doctors, so there was hesitation.

From the more junior staff members' perspective, the inclusion of staff in the WhatsApp group was the team leader’s decision alone. Seema (nurse), who praised Dr Manish for his leadership in setting up the group, but was not a part of the group, stated:

I have left it on the sir [Dr. Manish]. He might have felt that I am not required in the group and I also never said anything to him about it.

### Messages Sent in WhatsApp

Over the course of 18 weeks, 279 messages were exchanged in WhatsApp. Although 9 members sent messages, almost three-quarters of all messages were sent from the leader of the team or the coach. The majority (73.8%, 206/279) were text messages, 22.9% (64/279) were images, and the remaining messages were documents or videos. The images were photos of completed data forms, resource and reference materials, documentation of PDSA cycles, and physical spaces within the hospital. The frequency of messages followed a pattern, increasing when a coach site visit was nearing, which remained high immediately following a coaching visit, and then waning until the coach or team leader began scheduling the next site visit.

Much of the perceived value of WhatsApp was the ability to quickly disseminate information or ideas to a group of people:

If we have an idea in our mind, we can communicate easily and everyone comes to know about the issue together. You are in a group, you send information and you don’t have to tell everyone separately. You conveyed the message to all at one go.

Seema

It also provided a medium for clarifying clinical or QI guidance that was shared with the whole team at the same time (9 messages [3.2%, 9/279] containing materials on clinical guidance; 4 messages [1.4%, 4/279] containing materials on general QI guidance; and 14 messages [5.0%, 14/279] pertaining to the team’s improvement activity such as their aim, standard operating procedure, and flow chart). Flow charts were used by the team to help analyze the steps of carrying out IV procedures to identify where the aseptic technique was not being followed:

They were not understanding the flow chart, so Doctor made the flow chart again and sent in WhatsApp...Everything was discussed in [WhatsApp].

Anita

Finally, the app was used to share data on compliance with the aseptic technique, revealing the gaps in and raising awareness of compliance (26 messages [9.3%, 26/279] on the team’s data):

We came to know our mistakes, like 10 samples happened and we washed hands properly in 7 only and so on. Then there comes a time that if 10 pricks happened then among all 10 handwashing was followed properly. This increased awareness.

Dr Vijay

### Accessibility All the Time

Respondents both praised and critiqued WhatsApp for facilitating access at all times. The key benefit was being able to disseminate information without having to convene a meeting with all staff, which proved difficult with staff shifts and workloads. WhatsApp provided a medium for staff to both share ideas or information at any time and engage with the information at any time:

Sometimes one is not active and cannot listen properly in-person meetings but on WhatsApp he can at least read whenever he is feeling comfortable. If you are discussing something and someone is very tired, not able to pay attention at that time, they can always read the messages anytime they are comfortable.

Dr Manish

Despite these benefits of WhatsApp, being accessible all the time came with two notable drawbacks. Logistically, there was the expectation that facility staff use their own personal data packages to engage in the WhatsApp group, including downloading documents and watching videos. Additionally, being accessible all the time resulted in little separation or balance between work and personal life. One respondent stated she was able to remain up to date on work through the WhatsApp communication while she was on leave. She viewed remaining informed as an asset, but it did not allow her to separate herself from work while away.

### Engaging (or Not) in WhatsApp

Across respondents, it was generally agreed that there were between 15 and 20 members in the WhatsApp group, but only 3 to 5 active participants. Although the WhatsApp transcript indicated that 9 members shared messages, most messages came from the team leader and coach, highlighting the variable levels of participation.

Several reasons for not actively participating in the WhatsApp group were presented. The hierarchical structure of staffing contributed to fear and hesitation around being judged by peers and superiors for one’s remarks, comments, or suggestions:

So, they were taking it as an order from senior authorities and we have to follow it. Like if we are working and you have got information from senior authorities so generally you don’t question back, you just follow it and if they are having any problem so they prefer to discuss it personally rather than on group.

Dr Arun

The same respondent (Dr Arun) posited the inverse as well, that in a hypothetical group of nurses a new nurse:

...might open up or maybe a [nurse with 20 years of experience] might not open up because if she had done any mistake then she is under fear that everybody will get to know about that.

WhatsApp was also a means of maintaining a record of messages and a means of promoting accountability:

This gets documented. In personal meetings, people forgot whatever you said, it will never be documented but on WhatsApp you can see that what has been said and by whom. It is like committing yourself when you are. When you discuss personally then there are so many suggestions which are coming but none of them ever came on WhatsApp. So there, people can talk freely as nobody is making video of anybody that who I speaking, but when you are typing then it becomes a proof that you have said this and then it’s his responsibility to do that task.

Dr Manish

This sentiment, expressed by a senior staff person, encapsulated one reason why group members were reluctant to actively participate. According to Dr Vijay, they did not want to be held accountable for any specific suggestion or idea:

If you put anything there then you will be bound to do that.

Anay, the coach, in contrast, viewed WhatsApp as an *informal mode of communication* and did not perceive the enhanced accountability attribute of the technology.

Manjeet, echoed by Dr Arun and Kajal, offered another interpretation for the lack of responses, suggesting there was no value in contributing via WhatsApp when in-person meetings and discussion were going to take place, indicating that the real value of WhatsApp, in the professional setting, was to disseminate and share information rather than discuss.

Raman, Dr Nisha, and Seema went further, stating that WhatsApp was *irritating* and *worthless* because:

It consumes our time, like we are busy in our work and then we got a message and then we had to check it and have to give reply so it wastes our time. So our attention is on WhatsApp rather than on patient care.

Raman

Similarly, it was argued that there were so many messages being sent via WhatsApp that it was impossible to read them all. This was countered by other respondents who silenced their mobile devices so they were not notified of new messages:

[While] doing something for which I don’t require my phone to buzz, you can always put it on silent.

Dr. Nisha

It was suggested that had group members been told there was an expectation of discussion in the group, they would have participated more actively; however, that expectation was not clear. According to Anay, the technology was able to report who had received and opened a message, but it could not prompt someone to reply, so it was never clear if someone was not responding because they were not able to or did not want to engage.

Finally, Anay shared that language may have presented a barrier to participation. Some of the group members were not as confident in English, which was the dominant language used in WhastsApp communication.

In spite of the variable participation in the WhatsApp group, one respondent pointed to the documented improvements in clinical practice, noting that whether staff responded in the app was not essential if they were implementing improved practices in their patient care:

We used to discuss that it is working because we were seeing through WhatsApp that number of pricks are being counted daily so we were discussing that something has kicked on, something has happened. Positive discussions started and through WhatsApp group we were seeing that positive results are coming then we realized that we might not be active but things are happening really good and that gives us motivation. A famous saying that motivation is more important than inspiration.

Dr Vijay

### WhatsApp Versus Other Communication Methods

Although WhatsApp was viewed overall as a valuable resource for sharing information, it was not a replacement to in-person meetings and discussion, rather a supplement. In-person meetings were needed to discuss the documents shared via the app:

Face-to-face is of course the best. WhatsApp is like you are kicking a board but the board will not kick you back. We cannot underestimate the power of personal interaction. WhatsApp is like we can be aware of all things.

Dr Vijay

Things cannot be understood clearly on WhatsApp but in meetings we can discuss it in detail and as a result we can understand it much better as we are getting both theoretical and practical information clearly, because you will get to know about it completely when you are seeing and discussing those situations practically but in WhatsApp this is not possible.

Raman

In-person meetings were also viewed as essential for building team work, which could not be done via WhatsApp.

There was some discussion of the value of conference calls to share information, but this was generally agreed to be an ineffective communication medium due to scheduling, cost, the limitations of having a remotely delivered lecture, and the challenges of having equal participation if too many people were on the call. Anay viewed conference calls as a new mode of communication for health care staff; although WhatsApp was not new, it was *tapping into something already existing*.

### Coaching via WhatsApp

Anay, the coach, indicated that there were benefits of coaching via WhatsApp as well as drawbacks (Textbox 1). He was able to share materials on QI methods via WhatsApp, so he did not have to bring hard copies to in-person meetings and so team members could review materials in preparation for a coaching visit. WhatsApp also functioned as a coaching management tool for setting up visits, which was particularly helpful with the team, given the scheduling challenges they experienced. Documentation in WhatsApp allowed the coach to stay up to date on the activities and efficiently tailor coaching to that specific activity and team needs. The coach could also observe when enthusiasm appeared to be lagging among team members and purposively schedule a visit to re-energize the group. Finally, the coach was able to review data and observe that data collection was ongoing, but that the team was not able to learn from the data.

From the coach’s perspective, a limitation of using an exclusively in-person coaching approach was the inability to remain a part of the improvement process when not at the facility. Anay explained that with in-person coaching, the coach would visit the facility, help plan PDSA cycles, but then not know if the facility QI team followed through on the “do” phase and frequently would not “study” the impact of the change until the coach returned for another visit. Via WhatsApp, however, Anay could prompt the team to “do” and “study” by posing questions about the results to the group. Thus, WhatsApp provided an avenue to *keep them on track through small-scale testing and correcting them when I saw them going astray*. For example, one message Anay sent emphasized the importance of doing small PDSA cycles to test proposed changes, such as a checklist:

It helps to do things in small bits...We might learn as we use this tool and may want to modify it.

**Elements of coaching that were and were not made easier by using WhatsApp.**

Made easier by WhatsApp

- Sharing materials
- Scheduling site visits
- Identifying gaps in quality improvement (QI) knowledge
- Prompting action related to analyzing problems and testing changes
- Observing ongoing team dynamics

Made more difficult by WhatsApp

- Gaining initial understanding of team dynamics
- Facilitating learning from data
- Performing direct observation of and feedback on clinical practice

Following the test for the checklist, Anay reminded the group of the questions they need to think about to determine if the checklist was successful:

Excited to know the results of the first test! We are seeking answers to 1. What is the current performance level on different steps of asepsis? 2. How easy or difficult is it to fill the checklist? 3. Any side effects of this change in how we draw blood samples? And finally *What do we do next?*

Anay, WhatsApp chat transcript

Anay shared that WhatsApp is a good complement to in-person meetings, but getting to know the team can only happen in-person:

It’s very difficult to understand the team dynamics from a WhatsApp conversation, at least with a new team it is quite difficult. Now that I know these team members, who is who, even with the tone of conversation I can make out a lot about team dynamics.

Anay

Anita noted that the coach had on one or two occasions *lowered his support* but *in between through WhatsApp he used to be in touch*.

### Other Uses of WhatsApp

Outside of the application of WhatsApp to improve processes and systems of care, the messaging platform was used to communicate about other aspects of clinical care. Both nurses and doctors sent patients images and other information to colleagues for input on diagnosis and treatment. This form of communication was also used to confront some of the hierarchy present in the staffing structure.

One respondent, a nurse, recounted recent experiences in which a doctor was not physically present but did not agree with her diagnosis or suggestion for treatment. She was able to take a video or photograph of the patient and share it via WhatsApp with the doctor and receive confirmation on the diagnosis and treatment plan:

Sometimes doctors are not there in the room and caesarean is there and we are telling this happened, so he tells that now baby is fine how come it was caesarean? It did not come in front of him and you will explain everything but he will not believe because it was not in front of his eyes. After that we thought to make video at that time because it happened with me personally. Two days ago, one baby’s x-ray was bad we took the picture and sent because I thought the senior resident does not understand because he was new. He told me the x-ray is fine but I took the picture of the x-ray and sent it to sir and I told him that x-ray is not fine, do something. The video making thing is helpful because at least you are having some proof that you are telling right.

Anita

Other uses of WhatsApp included scheduling or discussing nonwork-related issues such as celebrations.

## Discussion

WhatsApp was applied as a communications platform by a QI team in a NICU in one hospital in India. An improvement team aimed to improve compliance with aseptic protocols to reduce the risk of nosocomial infections. Using WhatsApp, QI team members shared materials, data on compliance with protocols, and, to a lesser extent, changes to test.

The principal use of WhatsApp among this team was to disseminate information, which, although an integral part of QI, is only part of the process. Dynamic discussion among team members and with the coach is an essential step in analyzing data and generating changes to test. Yet, this medium did not facilitate sharing among this team.

Perceptions of quality communication among improvement team members can yield improved patient outcomes [13] and team cohesion [14]. Mobile technology can facilitate communication between health workers [15]. However, the impact of frequent messaging on workload and delivering services was a concern of some of our respondents and has been documented in other research [10]. It is also possible that through the frequent messages too much information was being shared, overloading team members. Such information and communication overload can negatively impact productivity [16] and should be managed to keep QI team members engaged in the improvement work. How team members managed the influx of messages (eg, using the silent function) may have influenced their perception of WhatsApp and its application in the improvement work. Related was the sense of always being available and engaged in work, which may create conflict between personal and professional lives or may allow for greater flexibility in both [17,18]. The expectation of always being available, therefore, is a double-edged sword.

Hierarchy played a role in the relationships between senior and junior residents and between doctors and nurses. This manifested itself in who was included in the WhatsApp group and their levels of participation and likely was present in other forms of interprofessional interaction; thus, it is possible that WhatsApp reinforced existing hierarchies. Nurses who feel like they contribute to decision making with the doctors are less likely to leave their jobs; conversely, nurses who do not feel they have positive relationships with doctors experience greater levels of professional stress [19,20]. It is important to move toward a culture of respect and equity among staff to improve job satisfaction and health worker retention. Mentoring or other similar supportive relationships may aid in flattening the hierarchical structure.

A benefit of WhatsApp was the ability to send patient images to other clinical staff for review and input on treatment approaches. There is mixed evidence on the utility of using websites and mobile technologies for transmitting images for diagnosis, review, and feedback from experts [6,15] and as a mechanism for building capacity of providers in low- and middle-income settings [21]. Although we did not ask about concerns of patient privacy, it should be taken into consideration when deciding whether and how to implement a messaging platform like WhatsApp in clinical services [22]. During the course of this activity, WhatsApp implemented end-to-end encryption, which may provide adequate security for maintaining patient privacy; however, the ethical and legal implications should be examined thoroughly.

WhatsApp not only facilitated communication among team members but was a useful, though limited, tool for QI coaching. Specifically, WhatsApp provided a real-time way of identifying when the team was having problems in teamwork and participation in the QI activity, problem and data analysis, and closing PDSA cycles. WhatsApp was very helpful in providing guidance on doing PDSA in real time. Establishing an improvement aim, forming a cohesive team, and analyzing data were much harder to support using a messaging platform and required in-person site visits. This study did not examine the sustainability of improvements made by the QI team, but we would expect that a coach could remotely inspire continued enthusiasm among a team without needing to visit in-person.

WhatsApp and other similar mobile messaging platforms have been underutilized and under-researched in LMICs to facilitate communication around health care improvement. Our study is a small-scale pilot that offers valuable insight, but does not offer evidence on the effectiveness or cost-effectiveness of these technologies. Thus, the implementation of mobile messaging platforms and other mHealth interventions needs to be scaled up and rigorously evaluated to better understand how these technologies impact health outcomes. This study was also limited to the WhatsApp-based communications and did not capture other formal or ad hoc communications that were part of the QI activity.

This exploration in the application of WhatsApp to aid in communication within a QI team shows the platform’s promise and highlights some areas of consideration before implementation. First, the decision to use a mobile communication app should be discussed and agreed upon by all team members, and all team members should be invited to participate equally. Building on this, issues of hierarchy within the staff and QI team structure should be addressed both in-person and via mobile technologies to better engage all staff in improvement activities. Second, the volume of information shared should be managed to allow staff to review and reflect on the information. Similarly, a culture of use should be fostered that creates expectations of participation even if not in real time and includes ground rules on appropriate and inappropriate use and patient privacy issues. Team leaders should keep track of participation and react if people are not engaging in discussion in WhatsApp. For coaching teams, other tools, such as short videos, should be prepared to aid in improving team work, using analysis tools, and analyzing data that could be shared in WhatsApp.

## Abbreviations

IV: intravenous
LMIC: low- and middle-income country
NICU: neonatal intensive care unit
PDSA: plan-do-study-act
QI: quality improvement
USAID: United States Agency for International Development

## References

[1] Thomas EJ (2011). Improving teamwork in healthcare: current approaches and the path forward. BMJ Qual Saf.

[2] Proudfoot J, Jayasinghe UW, Holton C, Grimm J, Bubner T, Amoroso C, Beilby J, Harris MF (2007). Team climate for innovation: what difference does it make in general practice?. Int J Qual Health Care.

[3] Vogelsmeier A, Scott-Cawiezell J (2011). Achieving quality improvement in the nursing home: influence of nursing leadership on communication and teamwork. J Nurs Care Qual.

[4] Leonard M, Graham S, Bonacum D (2004). The human factor: the critical importance of effective teamwork and communication in providing safe care. Qual Saf Health Care.

[5] World Health Organization (2011). mHealth: New horizons for health through mobile technologies.

[6] Hall CS, Fottrell E, Wilkinson S, Byass P (2014). Assessing the impact of mHealth interventions in low- and middle-income countries--what has been shown to work?. Glob Health Action.

[7] Labrique AB, Vasudevan L, Kochi E, Fabricant R, Mehl G (2013). mHealth innovations as health system strengthening tools: 12 common applications and a visual framework. Glob Health Sci Pract.

[8] Agarwal S, Perry HB, Long L, Labrique AB (2015). Evidence on feasibility and effective use of mHealth strategies by frontline health workers in developing countries: systematic review. Trop Med Int Health.

[9] Gadgets 360.

[10] Dorwal P, Sachdev R, Gautam D, Jain D, Sharma P, Tiwari AK, Raina V (2016). Role of WhatsApp messenger in the laboratory management System: a boon to communication. J Med Syst.

[11] Johnston MJ, King D, Arora S, Behar N, Athanasiou T, Sevdalis N, Darzi A (2015). Smartphones let surgeons know WhatsApp: an analysis of communication in emergency surgical teams. Am J Surg.

[12] Henry JV, Winters N, Lakati A, Oliver M, Geniets A, Mbae SM, Wanjiru H (2016). Enhancing the supervision of community health workers with WhatsApp mobile messaging: qualitative findings From 2 low-resource settings in Kenya. Glob Health Sci Pract.

[13] Arling PA, Abrahamson K, Miech EJ, Inui TS, Arling G (2014). Communication and effectiveness in a US nursing home quality-improvement collaborative. Nurs Health Sci.

[14] Mickan SM, Rodger SA (2005). Effective health care teams: a model of six characteristics developed from shared perceptions. J Interprof Care.

[15] Free C, Phillips G, Watson L, Galli L, Felix L, Edwards P, Patel V, Haines A (2013). The effectiveness of mobile-health technologies to improve health care service delivery processes: a systematic review and meta-analysis. PLoS Med.

[16] Karr-Wisniewski P, Lu Y (2010). When more is too much: operationalizing technology overload and exploring its impact on knowledge worker productivity. Comput Human Behav.

[17] Roy G (2016). Impact of mobile communication technology on the work life balance of working women - a review of discourses. JCMR.

[18] Wright KB, Abendschein B, Wombacher K, O’Connor M, Hoffman M, Dempsey M, Krull C, Dewes A, Shelton A (2014). Work-related communication technology use outside of regular work hours and the work life conflict: the influence of communication technologies on perceived work life conflict, burnout, job satisfaction, and turnover intentions. Manag Commun Q.

[19] Lakshman S (2016). Nurse turnover in India: factors impacting nurses’ decisions to leave employment. SAJHRM.

[20] Sharma P, Davey A, Davey S, Shukla A, Shrivastava K, Bansal R (2014). Occupational stress among staff nurses: controlling the risk to health. Indian J Occup Environ Med.

[21] Swanson JO, Plotner D, Franklin HL, Swanson DL, Lokomba BV, Lokangaka A, Sayury PI, Figueroa L, Garces A, Muyodi D, Esamai F, Kanaiza N, Mirza W, Naqvi F, Saleem S, Mwenechanya M, Chiwila M, Hamsumonde D, McClure EM, Goldenberg RL, Nathan RO (2016). Web-based quality assurance process drives improvements in obstetric ultrasound in 5 low- and middle-income countries. Glob Health Sci Pract.

[22] Adesina AO, Agbele KK, Februarie R, Abidoye AP, Nyongesa HO (2011). Ensuring the security and privacy of information in mobile health-care communication systems. S Afr J Sci.

